# Bis[2-(benzyl­amino)­pyridine-κ*N* ^1^]bis­(2-formyl­phenolato-κ^2^ *O*,*O*′)nickel(II)

**DOI:** 10.1107/S1600536811001425

**Published:** 2011-01-15

**Authors:** Kouassi Ayikoé, Ray J. Butcher, Yilma Gultneh

**Affiliations:** a Department of Chemistry, Howard University, 525 College Street NW, Washington, DC 20059, USA

## Abstract

In the title complex, [Ni(C_7_H_5_O_2_)_2_(C_12_H_12_N_2_)_2_], the Ni^II^ atom lies on a center of inversion and is coordinated in an octa­hedral geometry by two 2-(benzyl­amino)­pyridine (2-BAP) and two 2-formyl­phenolate ligands with the O-atom donors in the equatorial plane and the pyridine N atoms in axial positions. There are hydrogen-bonding inter­actions between the secondary amine H atom and the phenolate O atom, as well as C—H⋯O inter­actions, which result in the dihedral angle between the aromatic phenyl ring of the 2-formyl­phenolate moiety and the pyridine ring being 80.23 (4)°. In the packing, there are both C—H⋯π inter­actions, which link the mol­ecules into chains along the *b* axis, and offset π–π inter­actions involving both the pyridine and phenyl rings of the 2-BAP ligands [centroid–centroid distances = 4.0100 (8) Å for the pyridine rings and 3.6601 (8) and 4.8561 (8) Å for the phenyl rings].

## Related literature

For the structures of similar octa­hedral nickel complexes, see: Assey *et al.* (2010*a* ▶,*b* ▶); Butcher *et al.* (2009 ▶); Gultneh *et al.* (2008 ▶). For bond-length data, Allen *et al.* (1987 ▶).
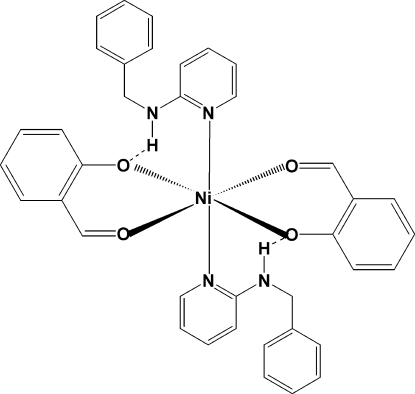


## Experimental

### 

#### Crystal data

[Ni(C_7_H_5_O_2_)_2_(C_12_H_12_N_2_)_2_]
*M* *_r_* = 669.40
Triclinic, 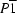

*a* = 8.1747 (5) Å
*b* = 9.3365 (5) Å
*c* = 10.9183 (6) Å
α = 73.926 (5)°
β = 84.766 (5)°
γ = 77.247 (5)°
*V* = 780.58 (8) Å^3^
*Z* = 1
Mo *K*α radiation
μ = 0.67 mm^−1^
*T* = 110 K
0.47 × 0.41 × 0.35 mm

#### Data collection

Oxford Diffraction Xcalibur diffractometer with a Ruby detector
Absorption correction: multi-scan (*CrysAlis PRO*; Oxford Diffraction, 2009 ▶) *T*_min_ = 0.932, *T*_max_ = 1.000
9822 measured reflections
5136 independent reflections
4216 reflections with *I* > 2σ(*I*)
*R*_int_ = 0.025

#### Refinement

*R*[*F* ^2^ > 2σ(*F* ^2^)] = 0.034
*wR*(*F* ^2^) = 0.086
*S* = 1.05
5136 reflections
218 parameters
H atoms treated by a mixture of independent and constrained refinement
Δρ_max_ = 0.46 e Å^−3^
Δρ_min_ = −0.27 e Å^−3^

### 

Data collection: *CrysAlis PRO* (Oxford Diffraction, 2009 ▶); cell refinement: *CrysAlis PRO*; data reduction: *CrysAlis PRO*; program(s) used to solve structure: *SHELXS97* (Sheldrick, 2008 ▶); program(s) used to refine structure: *SHELXL97* (Sheldrick, 2008 ▶); molecular graphics: *SHELXTL* (Sheldrick, 2008 ▶); software used to prepare material for publication: *SHELXTL*.

## Supplementary Material

Crystal structure: contains datablocks I, global. DOI: 10.1107/S1600536811001425/zl2338sup1.cif

Structure factors: contains datablocks I. DOI: 10.1107/S1600536811001425/zl2338Isup2.hkl

Additional supplementary materials:  crystallographic information; 3D view; checkCIF report

## Figures and Tables

**Table 1:** Hydrogen-bond geometry (Å, °) *Cg*4 is the centroid of the C1*A*–C6*A* ring.

*D*—H⋯*A*	*D*—H	H⋯*A*	*D*⋯*A*	*D*—H⋯*A*
N2*B*—H2*BN*⋯O1*A*	0.775 (17)	2.147 (18)	2.8550 (14)	152.0 (17)
C1*B*—H1*BA*⋯O1*A*^i^	0.95	2.42	2.9216 (14)	113
C3*B*—H3*BA*⋯*Cg*4^ii^	0.95	2.44	3.3674 (14)	166
C11*B*—H11*A*⋯*Cg*4^iii^	0.95	2.91	3.7535 (17)	148

Symmetry codes: (i) 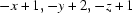; (ii) 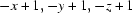; (iii) 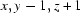.

**Fig. 1.**
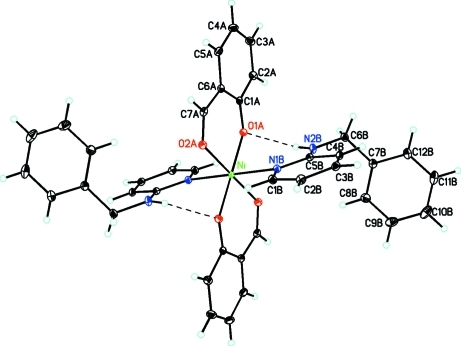
The molecular structure of the complex, C38H34N4NiO4, showing the atom numbering scheme and 50% probability displacement ellipsoids.

**Fig. 2.**
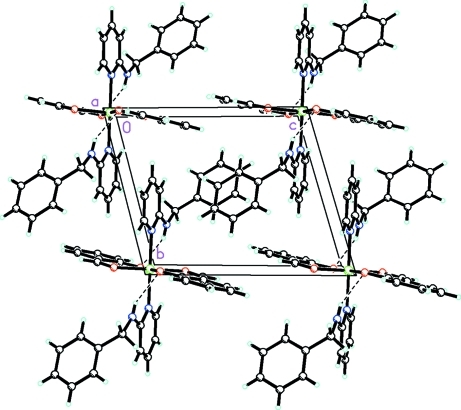
The molecular packing for C38H34N4NiO4, viewed down the c axis showing the intramolecular N—H···O interactions as dashed lines.

**Fig. 3.**
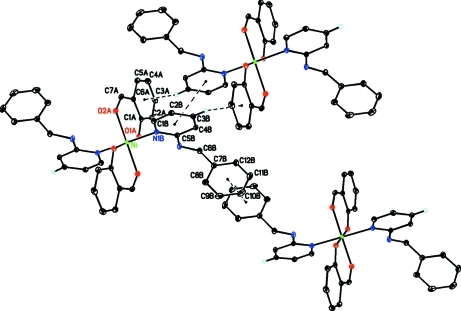
Molecular packing for C38H34N4NiO4 showing π–π and C-H···π interactions.

## supplementary crystallographic information

### Comment

As part of our continuing studies (Gultneh *et al.* 2008; Assey *et al.*, 2010*a*, 2010*b*; Butcher *et al.*, 2009) of nickel(II) complexes with relevance to the nickel containing metalloenzymes, we wish to report the structure of the mixed ligand complex, C_38_H_34_N_4_NiO_4_, where the Ni lies on a center of inversion and contains two 2-benzylaminopyridine (2-BAP) and two salicylaldehyde molecules where the O donors form an equatorial plane with pyridine N's in axial positions, thus the Ni is in an octahedral coordination environment.

The Ni—O and Ni—N bond distances (see Table 1) are within the normal ranges observed in other Ni complexes containing similar ligands (Allen *et al.*, 1987). There are hydrogen bonding interactions between the secondary amine H and phenolic O as well as C—H···O interactions which result in the dihedral angle between the aromatic phenyl ring of the salicylaldehyde moiety and the pyridine ring being 80.23 (4)°. The dihedral angle between the NiO_4_ plane and salicylaldehyde plane is 10.31 (5)°. In the packing there are both intermolecular C-H···π interactions (see Table  1) involving the salicylaldehyde anion which link the molecules into a chain in the b direction. In addition there are offsetting π–π interactions involving both the pyridine and phenyl rings of the 2-BAP moiety (see Figure 3). For the pyridine rings, Cg–Cg distance 4.0100 (8), perpendicular distance 3.3506 (5), slippage 2.203 Symmetry 1-x, 1-y, 1-z; for the phenyl rings (a) Cg-Cg -distance 3.6601 (8), perpendicular distance 3.4408 (5), slippage 1.248 Symmetry -x, 1-y, 2-z; (b) Cg–Cg distance 4.8561 (8), perpendicular distance 3.0743 (5), slippage 3.759 Symmetry 1-x, 1-y, 2-z.

### Experimental

Salicylaldehyde (0.23 g, 1.9 mmol) and 0.370 g of 2-benzylaminopyridine (2.0 mmol) were separately dissolved in 20 ml and 30 ml of methanol respectively before mixing and stirring under reflux followed by addition of 0.36 g (1.5 mmol) of NiCl_2_.6H_2_O in MeOH (20 ml). 2-Benzylaminopyridine, a secondary amine, has shown a chelating ability to nickel salts (nitrate and perchlorate) even in the presence of an aryl aldehyde (Butcher *et al.*, 2009). The solution of the salt and the two ligands was stirred overnight at room temperature. The mixture was evaporated under reduced pressure and a dark-green semi-solid was obtained. A small amount of the complex was then dissolved in 5 ml of DMF, filtered and layered with diethyl ether. Light green X-ray quality crystals were obtained after slow diffusion of the diethyl ether into DMF (yield 68%, m.p. 410 - 412 K). IR Data: 3330 cm^-1^ν(N—H) benzylamine nitrogen-hydrogen stretching; 3078 cm^-1^ and 3018 cm^-1^ν(C—H) aldehyde C—H stretching; 2851 cm^-1^ and 2770 cm^-1^ benzyl ν(C—H); 1615 cm^-1^ν(C=O) aldehyde carbonyl stretching; 1533 cm^-1^ and 1516 cm^-1^ν(N—C) and ν(N—H) bendings respectively; 1471 cm^-1^, 1445 cm^-1^ v(Ar C—H) bendings; 757 cm^-1^ν (out of plane aromatic bend). UV-vis data (in cm^-1^ with ε_max_ [*M*^-1^.cm^-1^] in brackets): 32154 (1847), 25510 (1306), 15476 (13), and 9174 (7). Room temperature magnetic moment was 3.01 BM.

### Refinement

H atoms were placed in geometrically idealized positions and constrained to ride on their parent atoms with C—H distances of 0.95 to 0.99 Å and *U*_iso_(H) = 1.2*U*_eq_(C). The positional and thermal parameters for the H atom attached to N were refined. The 0 2 0 reflection (which would have been the strongest reflection) was behind the beamstop and was omitted.

### Crystal data

**Table:**

[Ni(C_7_H_5_O_2_)_2_(C_12_H_12_N_2_)_2_]	*Z* = 1
*M**_r_* = 669.40	*F*(000) = 350
Triclinic, *P*1	*D*_x_ = 1.424 Mg m^−^^3^
Hall symbol: -P 1	Mo *K*α radiation, λ = 0.71073 Å
*a* = 8.1747 (5) Å	Cell parameters from 5541 reflections
*b* = 9.3365 (5) Å	θ = 4.8–32.6°
*c* = 10.9183 (6) Å	µ = 0.67 mm^−^^1^
α = 73.926 (5)°	*T* = 110 K
β = 84.766 (5)°	Block, pale green
γ = 77.247 (5)°	0.47 × 0.41 × 0.35 mm
*V* = 780.58 (8) Å^3^	

### Data collection

**Table:**

Oxford Diffraction Xcalibur diffractometer with a Ruby (Gemini Mo) detector	5136 independent reflections
Radiation source: Enhance (Mo) X-ray Source	4216 reflections with *I* > 2σ(*I*)
graphite	*R*_int_ = 0.025
Detector resolution: 10.5081 pixels mm^-1^	θ_max_ = 32.6°, θ_min_ = 4.8°
ω scans	*h* = −12→9
Absorption correction: multi-scan (*CrysAlis PRO*; Oxford Diffraction, 2009)	*k* = −14→13
*T*_min_ = 0.932, *T*_max_ = 1.000	*l* = −16→15
9822 measured reflections	

### Refinement

**Table:**

Refinement on *F*^2^	Primary atom site location: structure-invariant direct methods
Least-squares matrix: full	Secondary atom site location: difference Fourier map
*R*[*F*^2^ > 2σ(*F*^2^)] = 0.034	Hydrogen site location: inferred from neighbouring sites
*wR*(*F*^2^) = 0.086	H atoms treated by a mixture of independent and constrained refinement
*S* = 1.05	*w* = 1/[σ^2^(*F*_o_^2^) + (0.0457*P*)^2^] where *P* = (*F*_o_^2^ + 2*F*_c_^2^)/3
5136 reflections	(Δ/σ)_max_ < 0.001
218 parameters	Δρ_max_ = 0.46 e Å^−^^3^
0 restraints	Δρ_min_ = −0.27 e Å^−^^3^

### Special details

**Table:**

Geometry. All e.s.d.'s (except the e.s.d. in the dihedral angle between two l.s. planes) are estimated using the full covariance matrix. The cell e.s.d.'s are taken into account individually in the estimation of e.s.d.'s in distances, angles and torsion angles; correlations between e.s.d.'s in cell parameters are only used when they are defined by crystal symmetry. An approximate (isotropic) treatment of cell e.s.d.'s is used for estimating e.s.d.'s involving l.s. planes.
Refinement. Refinement of *F*^2^ against ALL reflections. The weighted *R*-factor *wR* and goodness of fit *S* are based on *F*^2^, conventional *R*-factors *R* are based on *F*, with *F* set to zero for negative *F*^2^. The threshold expression of *F*^2^ > σ(*F*^2^) is used only for calculating *R*-factors(gt) *etc*. and is not relevant to the choice of reflections for refinement. *R*-factors based on *F*^2^ are statistically about twice as large as those based on *F*, and *R*- factors based on ALL data will be even larger.

### Fractional atomic coordinates and isotropic or equivalent isotropic displacement parameters (Å^2^)

**Table:**

	*x*	*y*	*z*	*U*_iso_*/*U*_eq_	
Ni	0.5000	1.0000	0.5000	0.01289 (7)	
O1A	0.27528 (11)	1.00213 (9)	0.43885 (9)	0.01507 (18)	
O2A	0.61193 (11)	0.98245 (10)	0.32627 (9)	0.01674 (18)	
C1A	0.24508 (15)	0.97081 (12)	0.33607 (12)	0.0140 (2)	
C2A	0.07923 (16)	0.95941 (13)	0.31617 (13)	0.0172 (2)	
H2AA	−0.0068	0.9765	0.3782	0.021*	
C3A	0.04021 (17)	0.92416 (14)	0.20916 (14)	0.0213 (3)	
H3AA	−0.0721	0.9184	0.1990	0.026*	
C4A	0.16279 (18)	0.89655 (14)	0.11473 (14)	0.0221 (3)	
H4AA	0.1346	0.8713	0.0419	0.026*	
C5A	0.32436 (17)	0.90699 (14)	0.13020 (13)	0.0190 (3)	
H5AA	0.4081	0.8885	0.0670	0.023*	
C6A	0.36945 (16)	0.94477 (13)	0.23829 (12)	0.0150 (2)	
C7A	0.54127 (16)	0.95393 (13)	0.24225 (12)	0.0172 (2)	
H7A	0.6106	0.9358	0.1712	0.021*	
N1B	0.55584 (13)	0.75527 (11)	0.56185 (10)	0.0146 (2)	
N2B	0.28723 (13)	0.72549 (12)	0.63564 (11)	0.0173 (2)	
H2BN	0.262 (2)	0.8111 (19)	0.6004 (18)	0.026 (4)*	
C1B	0.71914 (16)	0.69158 (14)	0.54563 (13)	0.0166 (2)	
H1BA	0.7937	0.7570	0.5059	0.020*	
C2B	0.78339 (16)	0.53754 (14)	0.58336 (13)	0.0168 (2)	
H2BA	0.8998	0.4981	0.5729	0.020*	
C3B	0.67321 (16)	0.44103 (13)	0.63735 (12)	0.0165 (2)	
H3BA	0.7134	0.3339	0.6621	0.020*	
C4B	0.50697 (16)	0.50075 (13)	0.65462 (12)	0.0150 (2)	
H4BA	0.4310	0.4356	0.6915	0.018*	
C5B	0.44918 (15)	0.66092 (13)	0.61691 (12)	0.0139 (2)	
C6B	0.16081 (15)	0.63865 (14)	0.69494 (13)	0.0172 (2)	
H6BA	0.1547	0.5660	0.6453	0.021*	
H6BB	0.0503	0.7091	0.6912	0.021*	
C7B	0.19397 (15)	0.55083 (14)	0.83287 (13)	0.0167 (2)	
C8B	0.26795 (17)	0.60818 (16)	0.91340 (14)	0.0221 (3)	
H8BA	0.2980	0.7048	0.8822	0.027*	
C9B	0.29842 (19)	0.52611 (18)	1.03870 (15)	0.0295 (3)	
H9BA	0.3482	0.5672	1.0929	0.035*	
C10B	0.25672 (19)	0.38432 (18)	1.08552 (15)	0.0313 (3)	
H10A	0.2794	0.3275	1.1711	0.038*	
C11B	0.1819 (2)	0.32658 (16)	1.00657 (16)	0.0294 (3)	
H11A	0.1515	0.2302	1.0383	0.035*	
C12B	0.15109 (17)	0.40869 (15)	0.88107 (14)	0.0222 (3)	
H12A	0.1002	0.3677	0.8274	0.027*	

### Atomic displacement parameters (Å^2^)

**Table:**

	*U*^11^	*U*^22^	*U*^33^	*U*^12^	*U*^13^	*U*^23^
Ni	0.01303 (11)	0.01272 (11)	0.01305 (12)	−0.00388 (8)	−0.00051 (8)	−0.00262 (8)
O1A	0.0149 (4)	0.0147 (4)	0.0160 (4)	−0.0035 (3)	−0.0011 (3)	−0.0040 (3)
O2A	0.0159 (4)	0.0184 (4)	0.0162 (5)	−0.0050 (3)	−0.0002 (3)	−0.0040 (3)
C1A	0.0162 (6)	0.0083 (5)	0.0158 (6)	−0.0020 (4)	−0.0039 (5)	0.0001 (4)
C2A	0.0148 (6)	0.0146 (5)	0.0212 (6)	−0.0012 (5)	−0.0035 (5)	−0.0036 (5)
C3A	0.0193 (6)	0.0186 (6)	0.0268 (7)	−0.0031 (5)	−0.0088 (5)	−0.0055 (5)
C4A	0.0286 (7)	0.0193 (6)	0.0191 (7)	−0.0036 (5)	−0.0097 (6)	−0.0043 (5)
C5A	0.0246 (7)	0.0174 (5)	0.0139 (6)	−0.0046 (5)	−0.0023 (5)	−0.0017 (5)
C6A	0.0177 (6)	0.0123 (5)	0.0144 (6)	−0.0040 (5)	−0.0018 (5)	−0.0011 (4)
C7A	0.0193 (6)	0.0172 (5)	0.0137 (6)	−0.0048 (5)	0.0009 (5)	−0.0012 (5)
N1B	0.0143 (5)	0.0147 (4)	0.0150 (5)	−0.0045 (4)	0.0000 (4)	−0.0032 (4)
N2B	0.0144 (5)	0.0126 (5)	0.0219 (6)	−0.0035 (4)	0.0008 (4)	0.0005 (4)
C1B	0.0155 (6)	0.0175 (5)	0.0179 (6)	−0.0059 (5)	0.0017 (5)	−0.0050 (5)
C2B	0.0139 (6)	0.0184 (5)	0.0174 (6)	−0.0010 (5)	0.0014 (5)	−0.0060 (5)
C3B	0.0207 (6)	0.0136 (5)	0.0143 (6)	−0.0016 (5)	−0.0009 (5)	−0.0036 (4)
C4B	0.0177 (6)	0.0128 (5)	0.0140 (6)	−0.0047 (5)	0.0002 (5)	−0.0016 (4)
C5B	0.0148 (5)	0.0146 (5)	0.0127 (6)	−0.0042 (4)	−0.0009 (4)	−0.0032 (4)
C6B	0.0141 (6)	0.0182 (5)	0.0197 (6)	−0.0066 (5)	0.0000 (5)	−0.0032 (5)
C7B	0.0132 (5)	0.0170 (5)	0.0188 (6)	−0.0024 (5)	0.0024 (5)	−0.0043 (5)
C8B	0.0208 (6)	0.0264 (6)	0.0209 (7)	−0.0069 (5)	0.0020 (5)	−0.0086 (5)
C9B	0.0260 (7)	0.0431 (8)	0.0207 (7)	−0.0039 (7)	−0.0005 (6)	−0.0129 (7)
C10B	0.0258 (7)	0.0380 (8)	0.0187 (7)	0.0049 (6)	0.0035 (6)	0.0006 (6)
C11B	0.0301 (8)	0.0211 (6)	0.0287 (8)	−0.0029 (6)	0.0079 (7)	0.0023 (6)
C12B	0.0216 (6)	0.0186 (6)	0.0253 (7)	−0.0058 (5)	0.0031 (6)	−0.0040 (5)

### Geometric parameters (Å, °)

**Table:**

Ni—O1A	2.0052 (9)	N2B—H2BN	0.775 (17)
Ni—O1A^i^	2.0052 (9)	C1B—C2B	1.3755 (17)
Ni—O2A	2.0618 (9)	C1B—H1BA	0.9500
Ni—O2A^i^	2.0618 (9)	C2B—C3B	1.3920 (17)
Ni—N1B^i^	2.1509 (10)	C2B—H2BA	0.9500
Ni—N1B	2.1509 (10)	C3B—C4B	1.3676 (18)
O1A—C1A	1.2923 (15)	C3B—H3BA	0.9500
O2A—C7A	1.2434 (15)	C4B—C5B	1.4184 (16)
C1A—C2A	1.4237 (17)	C4B—H4BA	0.9500
C1A—C6A	1.4384 (18)	C6B—C7B	1.5187 (19)
C2A—C3A	1.3793 (18)	C6B—H6BA	0.9900
C2A—H2AA	0.9500	C6B—H6BB	0.9900
C3A—C4A	1.406 (2)	C7B—C8B	1.3878 (18)
C3A—H3AA	0.9500	C7B—C12B	1.3950 (17)
C4A—C5A	1.3745 (19)	C8B—C9B	1.385 (2)
C4A—H4AA	0.9500	C8B—H8BA	0.9500
C5A—C6A	1.4214 (17)	C9B—C10B	1.387 (2)
C5A—H5AA	0.9500	C9B—H9BA	0.9500
C6A—C7A	1.4311 (18)	C10B—C11B	1.381 (2)
C7A—H7A	0.9500	C10B—H10A	0.9500
N1B—C1B	1.3534 (16)	C11B—C12B	1.387 (2)
N1B—C5B	1.3594 (15)	C11B—H11A	0.9500
N2B—C5B	1.3507 (16)	C12B—H12A	0.9500
N2B—C6B	1.4497 (15)		
			
O1A—Ni—O1A^i^	180.000 (1)	C5B—N2B—H2BN	114.7 (13)
O1A—Ni—O2A	90.90 (4)	C6B—N2B—H2BN	120.9 (13)
O1A^i^—Ni—O2A	89.10 (4)	N1B—C1B—C2B	123.78 (11)
O1A—Ni—O2A^i^	89.10 (4)	N1B—C1B—H1BA	118.1
O1A^i^—Ni—O2A^i^	90.90 (4)	C2B—C1B—H1BA	118.1
O2A—Ni—O2A^i^	180.000 (1)	C1B—C2B—C3B	118.18 (11)
O1A—Ni—N1B^i^	88.39 (4)	C1B—C2B—H2BA	120.9
O1A^i^—Ni—N1B^i^	91.61 (4)	C3B—C2B—H2BA	120.9
O2A—Ni—N1B^i^	92.32 (4)	C4B—C3B—C2B	119.85 (11)
O2A^i^—Ni—N1B^i^	87.68 (4)	C4B—C3B—H3BA	120.1
O1A—Ni—N1B	91.61 (4)	C2B—C3B—H3BA	120.1
O1A^i^—Ni—N1B	88.39 (4)	C3B—C4B—C5B	119.29 (11)
O2A—Ni—N1B	87.68 (4)	C3B—C4B—H4BA	120.4
O2A^i^—Ni—N1B	92.32 (4)	C5B—C4B—H4BA	120.4
N1B^i^—Ni—N1B	180.00 (6)	N2B—C5B—N1B	117.55 (10)
C1A—O1A—Ni	127.16 (8)	N2B—C5B—C4B	121.45 (11)
C7A—O2A—Ni	123.83 (8)	N1B—C5B—C4B	120.99 (11)
O1A—C1A—C2A	119.19 (11)	N2B—C6B—C7B	113.76 (10)
O1A—C1A—C6A	124.31 (11)	N2B—C6B—H6BA	108.8
C2A—C1A—C6A	116.50 (11)	C7B—C6B—H6BA	108.8
C3A—C2A—C1A	121.71 (12)	N2B—C6B—H6BB	108.8
C3A—C2A—H2AA	119.1	C7B—C6B—H6BB	108.8
C1A—C2A—H2AA	119.1	H6BA—C6B—H6BB	107.7
C2A—C3A—C4A	121.58 (12)	C8B—C7B—C12B	118.46 (13)
C2A—C3A—H3AA	119.2	C8B—C7B—C6B	121.46 (11)
C4A—C3A—H3AA	119.2	C12B—C7B—C6B	120.08 (12)
C5A—C4A—C3A	118.48 (12)	C9B—C8B—C7B	120.72 (13)
C5A—C4A—H4AA	120.8	C9B—C8B—H8BA	119.6
C3A—C4A—H4AA	120.8	C7B—C8B—H8BA	119.6
C4A—C5A—C6A	121.73 (12)	C8B—C9B—C10B	120.44 (14)
C4A—C5A—H5AA	119.1	C8B—C9B—H9BA	119.8
C6A—C5A—H5AA	119.1	C10B—C9B—H9BA	119.8
C5A—C6A—C7A	116.26 (12)	C11B—C10B—C9B	119.35 (15)
C5A—C6A—C1A	119.98 (11)	C11B—C10B—H10A	120.3
C7A—C6A—C1A	123.76 (12)	C9B—C10B—H10A	120.3
O2A—C7A—C6A	128.67 (12)	C10B—C11B—C12B	120.30 (13)
O2A—C7A—H7A	115.7	C10B—C11B—H11A	119.9
C6A—C7A—H7A	115.7	C12B—C11B—H11A	119.9
C1B—N1B—C5B	117.88 (10)	C11B—C12B—C7B	120.72 (13)
C1B—N1B—Ni	114.15 (8)	C11B—C12B—H12A	119.6
C5B—N1B—Ni	127.95 (8)	C7B—C12B—H12A	119.6
C5B—N2B—C6B	123.33 (10)		
			
O2A—Ni—O1A—C1A	11.63 (9)	O1A—Ni—N1B—C5B	−37.62 (10)
O2A^i^—Ni—O1A—C1A	−168.37 (9)	O1A^i^—Ni—N1B—C5B	142.38 (10)
N1B^i^—Ni—O1A—C1A	103.93 (9)	O2A—Ni—N1B—C5B	−128.46 (10)
N1B—Ni—O1A—C1A	−76.07 (9)	O2A^i^—Ni—N1B—C5B	51.54 (10)
O1A—Ni—O2A—C7A	−11.59 (10)	C5B—N1B—C1B—C2B	−1.03 (19)
O1A^i^—Ni—O2A—C7A	168.41 (10)	Ni—N1B—C1B—C2B	177.25 (10)
N1B^i^—Ni—O2A—C7A	−100.02 (10)	N1B—C1B—C2B—C3B	2.3 (2)
N1B—Ni—O2A—C7A	79.98 (10)	C1B—C2B—C3B—C4B	−1.76 (19)
Ni—O1A—C1A—C2A	172.05 (8)	C2B—C3B—C4B—C5B	0.07 (19)
Ni—O1A—C1A—C6A	−7.43 (16)	C6B—N2B—C5B—N1B	−179.07 (11)
O1A—C1A—C2A—C3A	−179.07 (11)	C6B—N2B—C5B—C4B	0.13 (19)
C6A—C1A—C2A—C3A	0.45 (18)	C1B—N1B—C5B—N2B	178.42 (12)
C1A—C2A—C3A—C4A	0.5 (2)	Ni—N1B—C5B—N2B	0.40 (16)
C2A—C3A—C4A—C5A	−0.71 (19)	C1B—N1B—C5B—C4B	−0.78 (17)
C3A—C4A—C5A—C6A	−0.05 (19)	Ni—N1B—C5B—C4B	−178.80 (9)
C4A—C5A—C6A—C7A	−179.21 (12)	C3B—C4B—C5B—N2B	−177.92 (12)
C4A—C5A—C6A—C1A	1.01 (19)	C3B—C4B—C5B—N1B	1.25 (18)
O1A—C1A—C6A—C5A	178.32 (10)	C5B—N2B—C6B—C7B	63.67 (16)
C2A—C1A—C6A—C5A	−1.18 (17)	N2B—C6B—C7B—C8B	34.66 (16)
O1A—C1A—C6A—C7A	−1.45 (19)	N2B—C6B—C7B—C12B	−144.86 (12)
C2A—C1A—C6A—C7A	179.06 (11)	C12B—C7B—C8B—C9B	0.0 (2)
Ni—O2A—C7A—C6A	7.87 (18)	C6B—C7B—C8B—C9B	−179.53 (12)
C5A—C6A—C7A—O2A	−178.85 (12)	C7B—C8B—C9B—C10B	0.6 (2)
C1A—C6A—C7A—O2A	0.9 (2)	C8B—C9B—C10B—C11B	−1.0 (2)
O1A—Ni—N1B—C1B	144.30 (9)	C9B—C10B—C11B—C12B	0.9 (2)
O1A^i^—Ni—N1B—C1B	−35.70 (9)	C10B—C11B—C12B—C7B	−0.4 (2)
O2A—Ni—N1B—C1B	53.46 (9)	C8B—C7B—C12B—C11B	−0.1 (2)
O2A^i^—Ni—N1B—C1B	−126.54 (9)	C6B—C7B—C12B—C11B	179.45 (12)

Symmetry codes: (i) −*x*+1, −*y*+2, −*z*+1.

### Hydrogen-bond geometry (Å, °)

**Table:**

Cg4 is the centroid of the C1A–C6A ring.

**Table:**

*D*—H···*A*	*D*—H	H···*A*	*D*···*A*	*D*—H···*A*
N2B—H2BN···O1A	0.775 (17)	2.147 (18)	2.8550 (14)	152.0 (17)
C1B—H1BA···O1A^i^	0.95	2.42	2.9216 (14)	113
C3B—H3BA···Cg4^ii^	0.95	2.44	3.3674 (14)	166
C11B—H11A···Cg4^iii^	0.95	2.91	3.7535 (17)	148

Symmetry codes: (i) −*x*+1, −*y*+2, −*z*+1; (ii) −*x*+1, −*y*+1, −*z*+1; (iii) *x*, *y*−1, *z*+1.
